# Effect of repetitive transcranial magnetic stimulation with different stimulation parameters on post-stroke dysphagia: a systematic review and meta-analysis of randomized controlled trials

**DOI:** 10.3389/fneur.2025.1586734

**Published:** 2025-05-22

**Authors:** Xu-Miao Peng, Cheng Gong, Mei-Xia Xiao, Liang-Sheng Chen, Yi Li, Jie Chen, Mao-Yuan Wang, Yun Luo

**Affiliations:** ^1^College of Rehabilitation, Gannan Medical University, Ganzhou, China; ^2^Department of Rehabilitation Medicine, First Affiliated Hospital of Gannan Medical University, Ganzhou, China; ^3^Xiangya Hospital, Central South University, Changsha, China; ^4^The First Affiliated Hospital of Nanchang University, Nanchang City, Jiangxi Province, China; ^5^Department of Neurology, The First Affiliated Hospital of Army Medical University, Army Medical University, Chongqing, China; ^6^Ganzhou Intelligent Rehabilitation Technology Innovation Center, Ganzhou, China; ^7^Ganzhou Key Laboratory of Rehabilitation Medicine, Ganzhou, China

**Keywords:** stroke, dysphagia, repetitive transcranial magnetic stimulation, meta-analysis, randomized controlled trial

## Abstract

**Background:**

Previous studies have demonstrated the effectiveness of repetitive transcranial magnetic stimulation (rTMS) in treating post-stroke dysphagia (PSD). However, consensus on optimal clinical protocols for rTMS remains unclear. This study systematically evaluated the efficacy and safety of rTMS with different stimulation parameters in the treatment of PSD to provide evidence-based recommendations for clinical practice.

**Methods:**

Following Preferred Reporting Items for Systematic Reviews and Meta-Analysis (PRISMA) guidelines, related randomized controlled trials (RCTs) were searched across five databases (PubMed, Web of Science, Embase, Cochrane Library, MEDLINE) up to November 2024. Two reviewers independently screened studies, extracted data, and assessed quality using RevMan 5.40. Heterogeneity was evaluated via *I*^2^ values, with fixed/random effects models applied accordingly.

**Results:**

A total of 18 RCTs with 835 PSD patients were included in this study. The overall risk of bias in the included trials was evaluated as low, and the level of evidence recommendation was rated as “strong.” Meta-analyses demonstrated that both cerebral and cerebellar rTMS treatments significantly improved the swallowing function of PSD patients (*p* < 0.05). Subgroup analysis of cerebral rTMS showed that high-frequency rTMS (HF-rTMS) could effectively improve the swallowing function of PSD patients (*p* < 0.05), while low-frequency rTMS (LF-rTMS) failed to improve the swallowing function of PSD patients compared with the control group (*p* > 0.05). Furthermore, bilateral cerebral rTMS demonstrated superior efficacy in enhancing swallowing function compared to unilateral cerebral rTMS (*p* < 0.05). Subgroup analyses based on the Penetration Aspiration Scale (PAS) and Dysphagia Outcome Severity Scale (DOSS) revealed that cerebellar rTMS was more effective than cerebral rTMS in improving swallowing function in patients with PSD (*p* < 0.05). Regarding safety profiles, only 5 of the 18 RCTs documented mild and transient adverse events, including isolated cases of dizziness, headache, and temporary hearing impairment during treatment sessions.

**Conclusion:**

Both cerebral and cerebellar rTMS therapy can effectively and safely improve swallowing function in patients with PSD. Furthermore, cerebellar rTMS appears to be superior to cerebral rTMS in the treatment of PSD.

**Systematic review registration:**

https://www.crd.york.ac.uk/PROSPERO/view/CRD42024498567.

## 1 Background

Dysphagia is a common complication following stroke (1), with the incidence ranging from 37 to 78% (2, 3). Post-stroke dysphagia (PSD) frequently precipitates serious complications, including malnutrition, aspiration pneumonia, and electrolyte imbalances, which significantly impede rehabilitation efforts, affect quality of life, and potentially threaten survival (1, 4–6). Current conventional treatments for PSD encompass dietary modifications (7), postural adjustments (8), swallowing rehabilitation exercises (9–11), acupuncture (12, 13), and sensorimotor training (14–16). However, these interventions demonstrate limited efficacy and lack direct modulation of central nervous system governing swallowing function (17).

Repetitive transcranial magnetic stimulation (rTMS) is a non-invasive neuromodulation technique that modifies cortical excitability through repetitive magnetic pulses. Low-frequency rTMS (≤1 Hz; LF-rTMS) inhibits the cortical excitability, while high-frequency rTMS (>1 Hz; HF-rTMS) enhances the cortical excitability (18). rTMS has demonstrated significant advantages in treating PSD. Its non-invasive nature not only improves patient acceptance but also enhances treatment safety. Compared to traditional electrical stimulation therapies, rTMS does not require electrode attachment to the throat (as in neuromuscular electrical stimulation, NMES) or the use of invasive catheters (as in pharyngeal electrical stimulation, PES) (19, 20). Instead, it delivers stimulation non-invasively through the scalp, avoiding local adverse effects such as skin irritation or mucosal damage. Moreover, rTMS is particularly suitable for patients with severe dysphagia as it does not require active patient cooperation. From a safety perspective, rTMS has a low incidence of adverse effects (<5%), primarily manifesting as transient headaches or scalp discomfort, with severe complications such as seizures being extremely rare (18). In contrast, NMES and PES may induce discomfort such as laryngeal spasms or pain. More importantly, in terms of therapeutic mechanisms, traditional electrical stimulation therapies only target peripheral muscle groups, whereas rTMS directly modulates cortical activity in the brain’s swallowing functional areas, promoting neuroplasticity and functional reorganization. This helps restore central neural control over the pharyngeal muscles that have lost innervation (21). Additionally, rTMS offers greater precision and personalization in treatment planning. By measuring motor-evoked potential amplitudes to determine individualized stimulation thresholds, it enables precise control over stimulation intensity. This neurophysiology-based personalized treatment model, compared to the fixed-intensity protocols of traditional electrical stimulation therapies, is not only more scientifically rigorous but also allows for flexible parameter adjustments based on patient responses (22). Consequently, it enhances therapeutic efficacy while reducing the risk of adverse effects. Growing evidence from randomized controlled trials (RCTs) and meta-analyses supports the efficacy of rTMS in with the treatment of PSD (18, 23–30). Moreover, multiple meta-analyses further revealed that rTMS was more effective than transcranial direct current stimulation (tDCS), NMES, and PES in the treatment of PSD (31, 32).

Current research of rTMS for the treatment of PSD vary in stimulation site, frequency, intensity, and modality. For example, some studies showed positive outcomes in the treatment of cerebral rTMS (motor cortex associated with the mylohyoid muscle) (33, 34), while others reported comparable efficacy of cerebellar rTMS treatment (35). Bilateral or unilateral cerebral rTMS treatment also shows different effectiveness (28, 36). Frequency selection remains debated, with both LF-rTMS (37, 38) and HF-rMTS (28, 39, 40) demonstrating therapeutic benefits across studies. This parameter heterogeneity highlights the absence of standardized protocols in clinical practice. This meta-analysis systematically evaluated the efficacy and safety of rTMS with different stimulation parameters in the treatment of PSD, aiming to provide evidence-based recommendations for clinical practice.

## 2 Methods

This study was designed and implemented in accordance with the Preferred Reporting Items for Systematic Reviews and Meta-Analysis (PRISMA) guidelines (41). The study was registered in Prospero (CRD42024498567).

### 2.1 Retrieval strategy

For this study, the literature search was independently conducted by two researchers (L-SC and YiL). Five databases including Web of Science, PubMed, Embase, Cochrane Library, and MEDLINE, were searched for relevant studies from the establishment date of the databases until November 2024. The subject terms searched encompassed “Transcranial magnetic stimulation,” “Stroke” and “Dysphagia.” Relevant terms were retrieved through the above subject terms to further broaden the search. The following is an example of our search process in the PubMed database (Table 1).

**Table 1:** Search strategy for PubMed database.

No.	Search items
1	Stroke [MESH]
2	(Stroke*) OR (Cerebrovascular Accident*) OR (Cerebral Stroke*) OR (Stroke*, Cerebral) OR (Cerebrovascular Apoplexy) OR (Apoplexy, Cerebrovascular) OR (Vascular Accident, Brain) OR (Brain Vascular Accident*) OR (Vascular Accidents, Brain) OR (Cerebrovascular Stroke*) OR (Stroke*, Cerebrovascular) OR (Apoplexy) OR (CVA) OR (CVAs) OR (Cerebrovascular Accident) OR (Stroke*, Acute) OR (Acute Stroke*) OR (Cerebrovascular Accident*, Acute) OR (Acute Cerebrovascular Accident*)
3	#1 OR #2
4	Deglutition Disorders [MESH]
5	(Deglutition Disorder) OR (Disorders, Deglutition) OR (Dysphagia) OR (Swallowing Disorder*) OR (Oropharyngeal Dysphagia) OR (Dysphagia, Oropharyngeal) OR (Esophageal Dysphagia) OR (Dysphagia, Esophageal)
6	#4 OR #5
7	Transcranial Magnetic Stimulation [MESH]
8	(Magnetic Stimulation*, Transcranial) OR (Stimulation*, Transcranial Magnetic) OR (Transcranial Magnetic Stimulation*) OR (Transcranial Magnetic Stimulation, Paired Pulse) OR (Transcranial Magnetic Stimulation, Repetitive) OR (Transcranial Magnetic Stimulation, Single Pulse) OR (rTMS) OR (TMS) OR (TBS) OR (iTBS) OR (cTBS) OR (theta burst stimulation) OR (intermittent theta burst stimulation) OR (continuous theta burst stimulation)
9	#7 OR #8
10	#3 AND #6 AND #9

### 2.2 Inclusion and exclusion criteria of study

We conducted the literature screening based on pre-defined inclusion and exclusion criteria. The inclusion criteria were as follows: (1) Patients with PSD; (2) The primary intervention was rTMS; (3) The control group received sham stimulation or conventional swallowing intervention; (4) The primary outcome indicator was the assessment of swallowing function; (5) The study was an RCT; (6) The study published in English. The exclusion criteria were: (1) Animal experiments; (2) Duplicated data; (3) Inability to obtain the full text; (4) Missing data and inability to extract complete data.

### 2.3 Study selection

All retrieved literature was imported into the EndNote 20 reference management software, where duplicate studies were systematically eliminated using the built-in deduplication tool. Two independent reviewers (CG and X-MP) conducted parallel title/abstract screening against predefined inclusion/exclusion criteria. For studies meeting preliminary eligibility requirements, full-text articles were retrieved and subjected to comprehensive evaluation. Discrepancies in screening decisions were resolved through consultation with the principal investigator (YuL), with final determinations made by consensus.

### 2.4 Data extraction

Two reviewers (CG and X-MP) independently extracted the following parameters from included studies: (1) Bibliometric data: first author, publication year and sample size; (2) Demographic characteristics: sex, age distribution, stroke classification (ischemic/hemorrhagic), lesion localization and disease duration; (3) Intervention protocols: stimulation site coordinates, intensity, frequency (Hz), pulse count per session and total intervention duration. During data extraction, discrepancies or incomplete records were resolved through correspondence with original authors. Studies with unresolved data gaps after three unsuccessful contact attempts were classified as containing incomplete datasets. Inter-rater disagreements were adjudicated by consultation with the principal investigator (YuL) to establish methodological consensus.

### 2.5 Quality assessment

The quality assessment of included studies was independently conducted by two researchers (CG and X-MP) through a standardized process: initial individual evaluations followed by consensus discussions. The risk of bias assessment was performed using the Cochrane Risk of Bias Tool in Revman 5.40 (42), with results visually represented through a three-tier color-coded system: high risk (red), unclear risk (yellow), and low risk (green). Statistical heterogeneity analysis among studies was conducted using Revman 5.40, with *I*^2^ values quantifying heterogeneity levels: ≥75% (high heterogeneity), 50–75% (moderate heterogeneity), 25–50% (low heterogeneity), and when *I*^2^ = 0%, the heterogeneity was judged as no heterogeneity (43).

The quality of evidence for the outcome indicators was evaluated using the Grading of Recommendations Assessment, Development, and Evaluation (GRADE) system, which examines aspects such as the limitations, indirectness, inconsistency, and imprecision of the studies (44). The results were evaluated by grading the evidence for the outcome indicators as “high,” “moderate,” “low,” or “very low.” The strength of the recommendations was classified into two levels: “strong” or “weak” (45).

### 2.6 Statistical analysis

Statistical analyses were performed using Revman 5.40 with the extracted datasets. For meta-analysis implementation, model selection followed heterogeneity thresholds: random-effects models were applied when *I*^2^ ≥ 50%, while fixed-effects models were adopted for *I*^2^ < 50% cases. Mean Deviation (MD) and 95% Confidence Interval (CI) were used to represent the magnitude of the effect size of the research results.

## 3 Results

### 3.1 Literature search findings

**Figure 1:**
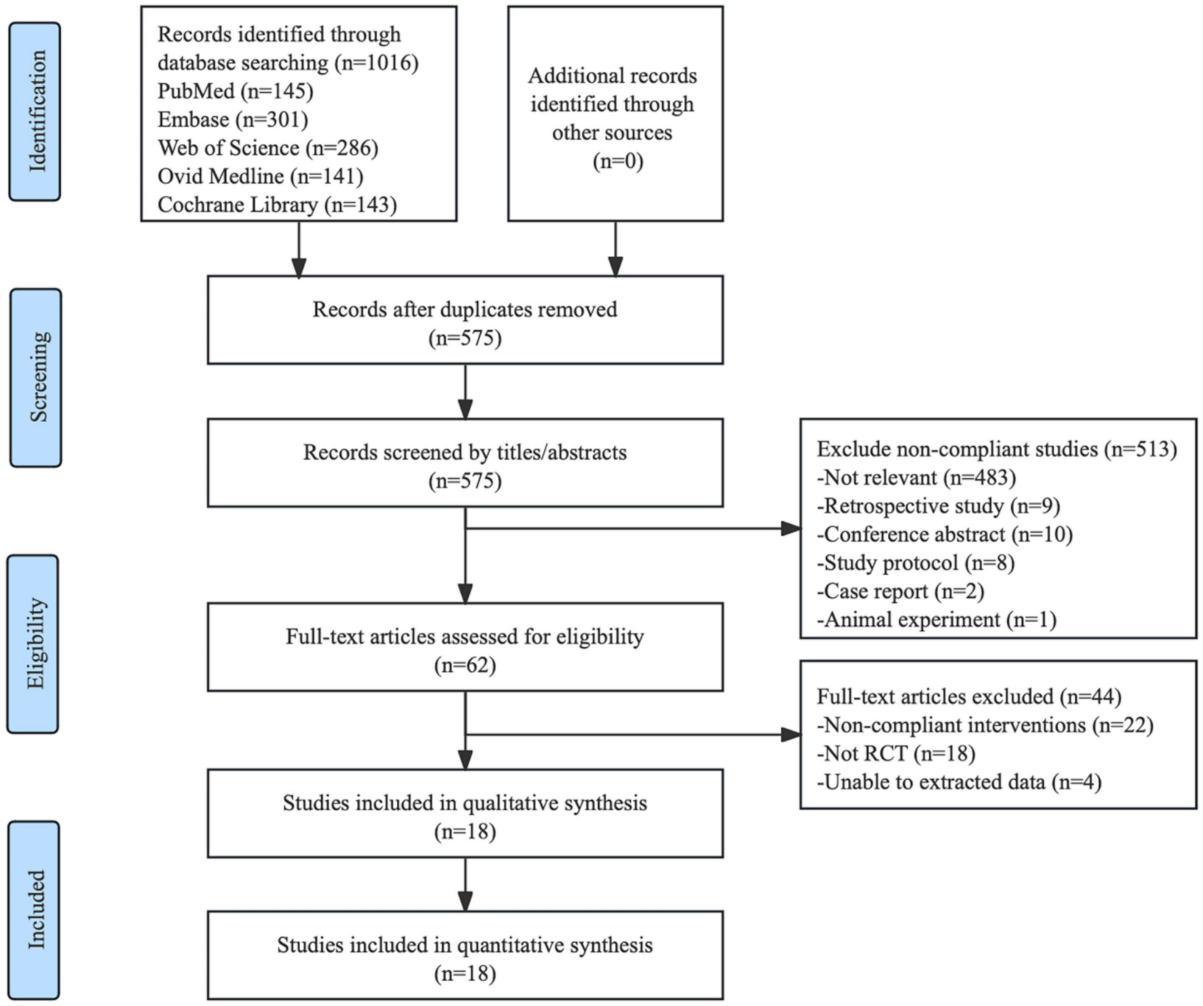
Literature search flowchart.

A total of 1,016 records were identified through database searches. Following the management of EndNote 20 reference software, 441 duplicates were automatically removed through its deduplication feature. Two independent reviewers conducted title/abstract screening, excluding 513 records based on predefined inclusion/exclusion criteria. The remaining 62 potentially relevant articles underwent full-text evaluation, with exclusions comprising: (1) 18 non-randomized controlled trials; (2) 22 studies employing non-compliant interventions; (3) 4 publications with incomplete datasets. This rigorous process yielded 18 eligible RCTs for further analysis (Figure 1).

### 3.2 Characteristics of included studies

The 18 included RCTs (27, 28, 33, 37–40, 46–56) encompassed 835 participants with sample sizes ranging from 28 to 143. Of them, there were 515 males and 320 females with PSD. The characteristics of these studies were systematically documented, including sample demographics, stroke classification, lesion topography, disease duration, and validated outcome measures: Penetration Aspiration Scale (PAS), Standardized Swallowing Assessment (SSA), Water Swallow Test (WST), Functional Oral Intake Scale (FOIS), Fiberoptic Endoscopic Dysphagia Severity Scale (FEDSS), Degree of Dysphagia (DD), Videofluoroscopic Dysphagia Scale (VDS), and Dysphagia Outcome Severity Scale (DOSS) (Table 2).

**Table 2:** Clinical characteristics of included studies.

Study (year)	Sample size	Sex (M/F)	Age (year)	Duration of disease	Stroke type	Region of lesion	Outcome	Adverse effects
Rao et al., 2022 (49)	G1: 33 G2: 31	20/11 24/7	63.42 ± 10.35 65.9 ± 11.42	21.29 ± 12.40 D 23.15 ± 12.44 D	Hemorrhagic (30) Ischemic (34)	Cortex (10) Subcortical (31) Brainstem (8) Multiple (15)	FEDSS PAS SSA FOIS	None
Dai et al., 2023 (46)	G1: 14 G2: 14 G3:14	11/3 12/2 12/2	61.50 ± 1.94 59.86 ± 3.91 58.93 ± 3.07	78.68 ± 47.58 D 62.33 ± 68.78 D 69.60 ± 62.19 D	Hemorrhagic (5) Ischemic (37)	Cerebellum (1) Brainstem (33) Both (8)	PAS FOIS DOSS	None
Zhong et al., 2023 (51)	G1: 41 G2: 43	24/17 21/22	63.61 ± 9.67 62.81 ± 11.49	22.06 ± 11.52 D 30.00 ± 19.94 D	Hemorrhagic (31) Ischemic (53)	-	PAS FEDSS	None
Dong et al., 2022 (56)	G1: 12 G2: 11 G3:11	6/6 7/4 6/5	49.67 ± 11.285 4.18 ± 10.54 57.55 ± 8.57	25.50 ± 9.28 D 21.00 ± 5.70 D 24.91 ± 6.89 D	Hemorrhagic (4) Ischemic (30)	Pons (26) Medulla Oblongata (4) Multiple Brainstem (4)	PAS	Headache
Wang et al., 2023 (55)	G1: 11 G2: 10 G5:10 G3: 10 G4: 10 G6:10	7/4 6/4 7/3 6/4 6/4 7/3	57.72 ± 8.36 58.10 ± 8.24 57.91 ± 8.72 58.92 ± 8.47 59.31 ± 8.53 59.40 ± 8.42	8.31 ± 3.92 W 8.79 ± 3.67 W 8.57 ± 3.78 W 8.91 ± 3.83 W 8.96 ± 3.77 W 8.82 ± 3.75 W	Hemorrhagic (20) Ischemic (41)	Cortical (3) Subcortical (32) Both (26)	PAS SSA DOSS	Headache Dizzy
Tai et al., 2023 (50)	G1: 15 G2: 15 G5:15 G3: 15 G4: 15 G6:15	9/6 6/9 7/8 10/5 10/5 9/6	56.67 ± 13.31 60.13 ± 15.10 57.07 ± 16.87 60.12 ± 9.98 53.00 ± 11.84 59.25 ± 12.66	3.54 ± 2.15 M 3.93 ± 1.97 M 3.20 ± 1.83 M 3.72 ± 1.94 M 3.75 ± 1.53 M 3.75 ± 1.92 M	Hemorrhagic (42) Ischemic (48)	Supratentorial (45) Infratentorial (45)	SSA	None
Suh et al., 2024 (54)	G1: 14 G2: 14	7/7 9/5	64.93 ± 16.60 68.64 ± 12.83	63.36 ± 49.88 D 68.71 ± 43.32 D	Hemorrhagic (9) Ischemic (19)	-	PAS	-
Zhong et al., 2021 (53)	G1: 38 G2: 36 G3: 34 G4: 35	28/10 28/8 20/14 18/17	64.47 ± 13.95 64.67 ± 10.87 63.18 ± 9.92 62.34 ± 11.54	35.33 ± 34.67 D 31.51 ± 35.52 D 21.51 ± 12.19 D 23.22 ± 11.59 D	Hemorrhagic (54) Ischemic (89)	-	FEDSS PAS SSA	Headache
Khedr et al., 2009 (48)	G1: 14 G2: 12	10/16	56.9 ± 11.7 56.2 ± 13.4	-	Ischemic (26)	Cortical (13) Subcortical (3) Both (10)	DD	-
Khedr et al., 2010 (47)	G1: 11 G2: 11	8/3 8/3	56.7 ± 16 55.4 ± 9.7	6.0 ± 4.2 W 5.5 ± 0.2 W	Ischemic (22)	Lateral medullary (11) Brainstem (11)	DD	-
Park et al., 2013 (39)	G1: 9 G2: 9	5/4 5/4	73.7 ± 15.1 58.9 ± 13.4	59.9 ± 16.3 D 63.9 ± 26.8 D	Hemorrhagic (3) Ischemic (15)	Middle cerebrum (16) Basal ganglia (1) Striatocapsular (1)	PAS VDS	-
Lim et al., 2014 (37)	G1: 20 G2: 14	9/11 6/8	61.8 ± 10.4 59.8 ± 11.8	32.0 ± 13.9 D 30.3 ± 14.8 D	Hemorrhagic (24) Ischemic (10)	-	PAS	Headache
Zou et al., 2023 (40)	G1: 15 G2: 15	6/9 10/5	59.33 ± 6.85 60.07 ± 5.40	20.07 ± 5.06 D 20.13 ± 5.68 D	Hemorrhagic (11) Ischemic (19)	-	PAS FOIS	None
Du et al., 2016 (27)	G1:15 G2:13 G3:12	13/2 7/6 6/6	58.20 ± 2.78 57.92 ± 2.47 58.83 ± 3.35	< 2 M	Ischemic (40)	Cortical (3) Subcortical (24) Multiple (13)	WST SSA DD	None
Park et al., 2017 (28)	G1:11 G2:11 G3:11	8/3 8/3 7/4	60.2 ± 13.8 67.5 ± 13.4 69.6 ± 8.6	4.1 ± 2.4 W 4.2 ± 1.7 W 6.6 ± 7.8 W	Hemorrhagic (10) Ischemic (23)	Supratentorial (29) Infratentorial (4)	PAS DOSS VDS	None
Tarameshlu et al., 2019 (33)	G1:6 G2:6	1/5 4/2	74.67 ± 5.92 66.00 ± 5.55	5.33 ± 3.38 M 3.17 ± 1.72 M	-	Cortical (8) Subcortical (4)	FOIS	-
Ünlüer et al., 2019 (38)	G1:15 G2:13	6/9 6/7	67.80 ± 11.88 69.31 ± 12.89	105.9 ± 49.0 D 101.4 ± 42.0 D	Hemorrhagic (2) Ischemic (26)	-	PAS	Headache Hearing loss Dizzy Nosebleed
Wen et al., 2022 (36)	G1:18 G2:20	13/5 16/4	66.28 ± 10.42 67.00 ± 8.43	0.87 ± 0.75 M 0.62 ± 0.51 M	Ischemic (38)	-	FEDSS PAS SSA FOIS	None

G1, group 1; G2, group 2; G3, group 3; G4, group 4; G5, group 5; G6, group 6; M/F, Male/Female; “-”, not recorded; D, days; W, weeks; M, months; PAS, Penetration Aspiration score; FOIS, Functional Oral Intake Scale; WST, Water Swallow Test; FEDSS, Fiberoptic Endoscopic Dysphagia Severity Scale; SSA, Standardized Swallowing Assessment; DD, Degree of Dysphagia; VDS, Videofluoroscopic Dysphagia Scale; DOSS, Dysphagia Outcome Severity Scale.

Heterogeneity was observed in rTMS protocols across studies (Table 3). Stimulation parameters varied in (1) stimulation site: cerebral mylohyoid cortex (14 RCTs) vs. cerebellum (5 RCTs); (2) lateralization: unilateral (11 RCTs) vs. bilateral (4 RCTs) approaches, with 3 RCTs combining both; (3) stimulation frequency: HF-rTMS (3 Hz, 5 Hz, 10 Hz, 50 Hz; 13 RCTs) vs. LF-rTMS (1 Hz; 3 RCTs), including 2 RCTs with dual-frequency paradigms.

**Table 3:** The stimulation parameters of rTMS.

Study (year)	Stimulation site	Intensity (%RMT)	Frequency (Hz)	Number of pulses (times)	Number of sessions (day)	Duration of intervention
Rao et al., 2022 (49)	Bilateral cerebellum	100	50	600	10	2w, 5d/w
Dai et al., 2023 (46)	G1: Ipsilateral cerebellum G2: Bilateral cerebellum	90	10	500	10	2w, 5d/w
Zhong et al., 2023 (51)	Bilateral cerebellum	80	10	500	10	2w, 5d/w
Dong et al., 2022 (56)	G1: Bilateral cerebellum G2: Ipsilateral cerebellum	80	10	500	10	2w, 5d/w
Wang et al., 2023 (55)	Contralateral	100	5 (G1, G3) 1 (G2, G4)	950 (G1, G3) 1,005 (G2, G4)	10	2w, 5d/w
Tai et al., 2023 (50)	Contralateral (G1, G3) Ipsilateral (G2, G4)	80	50	600	20	4w, 5d/w
Suh et al., 2024 (54)	Ipsilateral	80% AMT	50	600	5	1w, 5d/w
Zhong et al., 2021 (53)	G1: Contralateral G2: Ipsilateral G3: Cerebellum	110	5	1800	10	2w, 5d/w
Khedr et al., 2009 (48)	Ipsilateral	120	3	300	5	1w, 5d/w
Khedr et al., 2010 (47)	Bilateral cerebrum	130	3	300	5	1w, 5d/w
Du et al., 2016 (27)	G1: Ipsilateral G2: Contralateral	90 100	3 1	1,200	5	1w, 5d/w
Park et al., 2013 (39)	Contralateral	90	5	500	10	2w, 5d/w
Lim et al., 2014 (37)	Contralateral	100	1	1,200	10	2w, 5d/w
Zou et al., 2023 (40)	Bilateral cerebrum	90	5	1,200	20	4w, 5d/w
Park et al., 2017 (28)	G1: Bilateral cerebrum G2: Ipsilateral	90	5	1,000	10	2w, 5d/w
Tarameshlu et al., 2019 (33)	Contralateral	120	1	1,200	5	1w, 5d/w
Ünlüer et al., 2019 (38)	Contralateral	90	1	1,200	15	3w, 5d/w
Wen et al., 2022 (36)	Ipsilateral	120	5	1800	20	4w, 5d/w

Ipsilateral/Contralateral: motor cortex associated with the mylohyoid muscle of the affected/unaffected cerebral hemisphere; G1, group 1; G2, group 2; G3, group 3; G4, group 4; RMT, resting motor threshold; AMT, active motor threshold; D, day; W, week.

### 3.3 Quality assessment result

**Figure 2:**
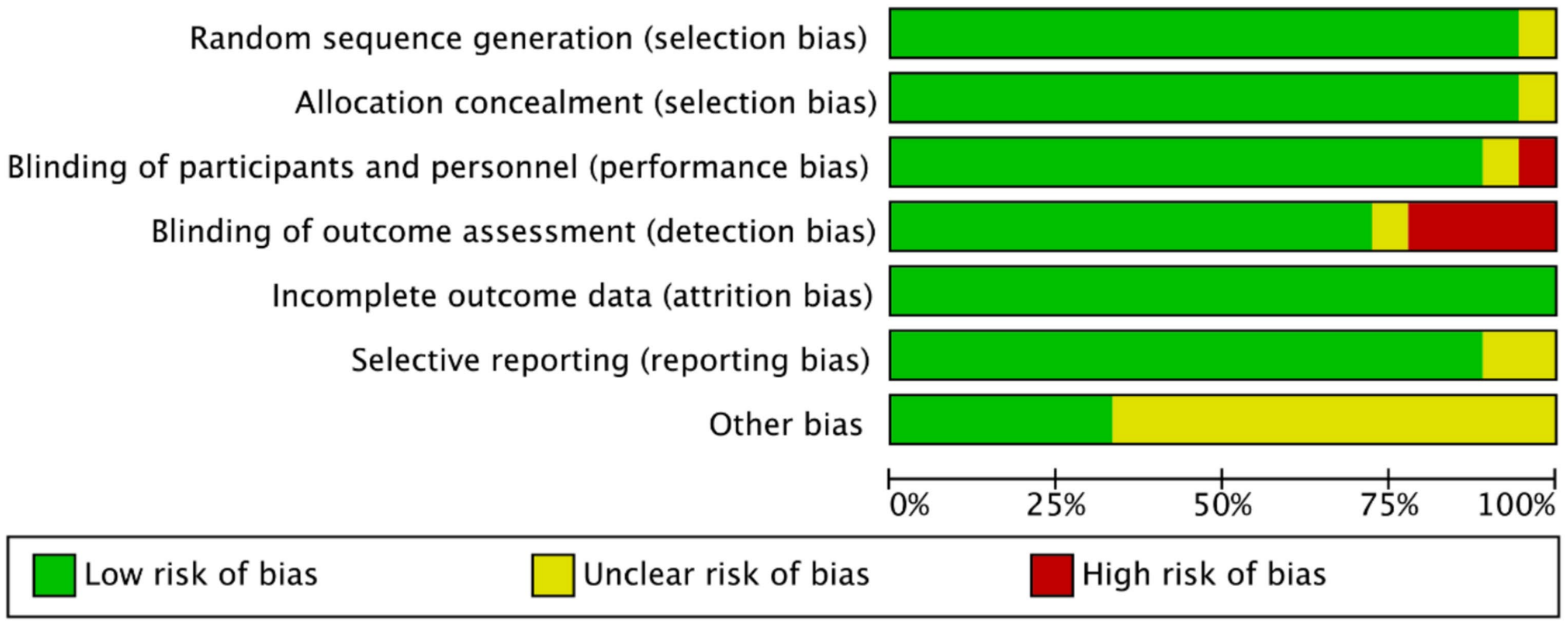
Risk of bias map of included studies.

**Figure 3:**
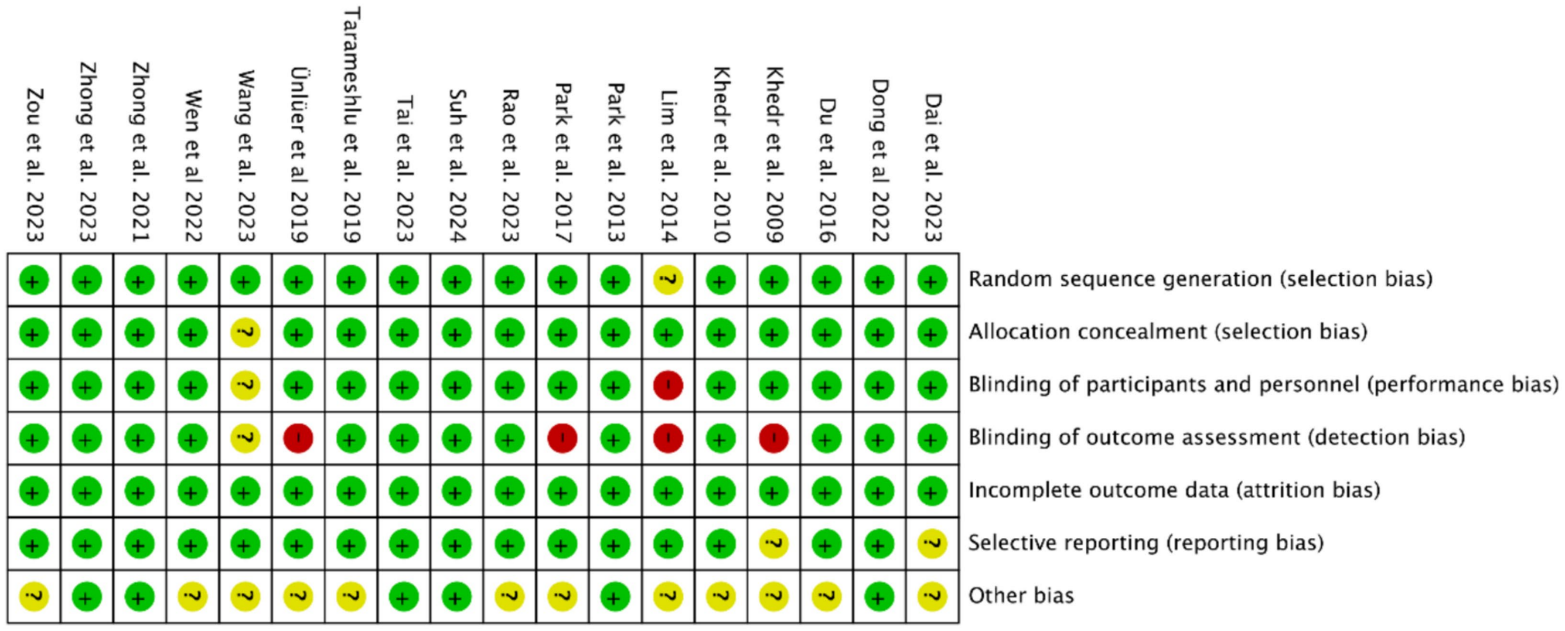
Summary of risk of bias of included studies.

Methodological quality assessment was performed using the Cochrane Risk of Bias Tool in Revman 5.40. Critical appraisal revealed: (1) Inadequate participant/personnel blinding in 1 study (elevated performance bias risk); (2) Absence of outcome assessor blinding in four studies (detection bias concerns); (3) Unclear selective reporting risks in three studies due to incomplete outcome data disclosure. Notwithstanding these limitations, the majority of included trials demonstrated low overall bias risk (Figures 2, 3).

The GRADE evaluation of primary outcomes identified downgrades in evidence certainty for the WST and VDS metrics, attributed to insufficient sample sizes (*n* < 100), which led to serious imprecision ratings. Additionally, while the DD scale is clinically convenient and widely adopted, its reliability and validity remain understudied in high-quality research, further diminishing its evidence grade as an informal assessment tool. Consequently, these outcomes were classified as “moderate” (Table 4). In contrast, five swallowing-related measures (PAS, DOSS, FEDSS, SSA, FOIS) maintained “high” ratings, and thus the final evidence recommendation level was “strong.”

**Table 4:** GRADE level of evidence rating scale for indicators of consequences.

Certainty assessment							No. of participants				Effect
No. of studies	Study design	Risk of bias	Inconsistency	Indirectness	Imprecision	Publication bias	Experimental group	Control group	SMD [95% CI]	Size	Certainty
Level of PSD											
Outcome: PAS											
13	RCT	Not serious	Not serious	Not serious	Not serious	Not serious	*n* = 351	*n* = 340	−1.64 [−2.19, −1.09]	Moderate	⊕ ⊕ ⊕⊕ High
Outcome: DOSS											
3	RCT	Not serious	Not serious	Not serious	Not serious	Not serious	*n* = 91	*n* = 90	0.99[0.48, 1.50]	Moderate	⊕ ⊕ ⊕⊕ High
Outcome: WST											
1	RCT	Not serious	Not serious	Not serious	Not serious	Not serious	*n* = 28	*n* = 24	−1.48[−2.46, −0.50]	Little	⊕ ⊕ ⊕⊖ Moderate
Outcome: FEDSS											
4	RCT	Not serious	Not serious	Not serious	Not serious	Not serious	*n* = 164	*n* = 165	−0.47 [−0.79, −0.14]	Moderate	⊕ ⊕ ⊕⊕ High
Outcome: SSA											
6	RCT	Not serious	Not serious	Not serious	Not serious	Not serious	*n* = 252	*n* = 246	−1.76 [−2.63, −0.90]	Moderate	⊕ ⊕ ⊕⊕ High
Outcome: DD											
3	RCT	Not serious	Not serious	Not serious	Not serious	Not serious	*n* = 53	*n* = 47	−1.37[−1.79, −0.95]	Moderate	⊕ ⊕ ⊕⊖ Moderate
Outcome: VDS											
2	RCT	Not serious	Not serious	Not serious	Not serious	Not serious	*n* = 31	*n* = 31	−9.19 [−21.03,2.65]	Little	⊕ ⊕ ⊕⊖ Moderate
Outcome: FOIS											
4	RCT	Not serious	Not serious	Not serious	Not serious	Not serious	*n* = 75	*n* = 85	0.80[−0.32,1.29]	Moderate	⊕ ⊕ ⊕⊕ High

### 3.4 Efficacy of cerebral rTMS

**Figure 4:**
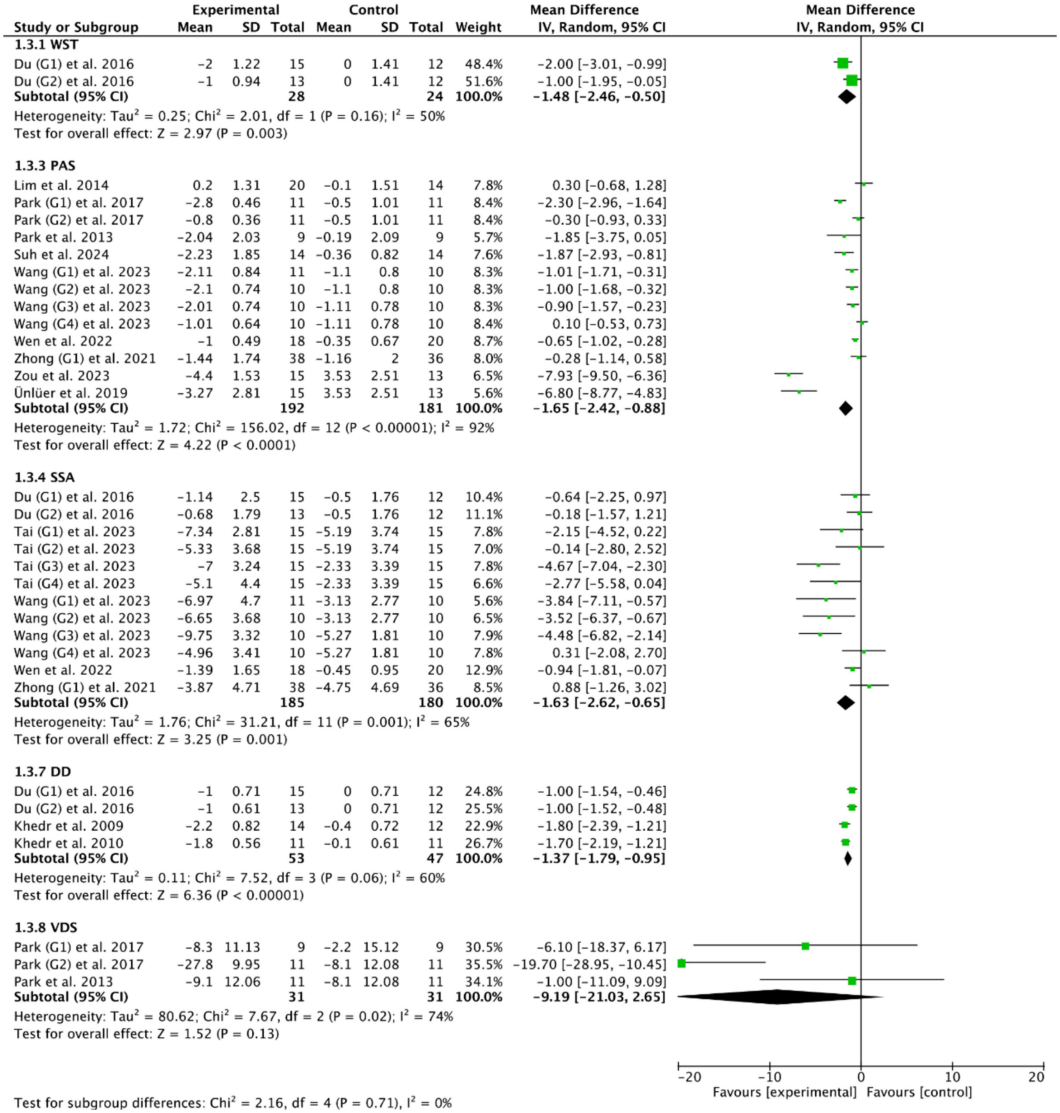
Efficacy of cerebral rTMS evaluated by the WST, PAS, SSA, DD, and VDS scales.

**Figure 5:**
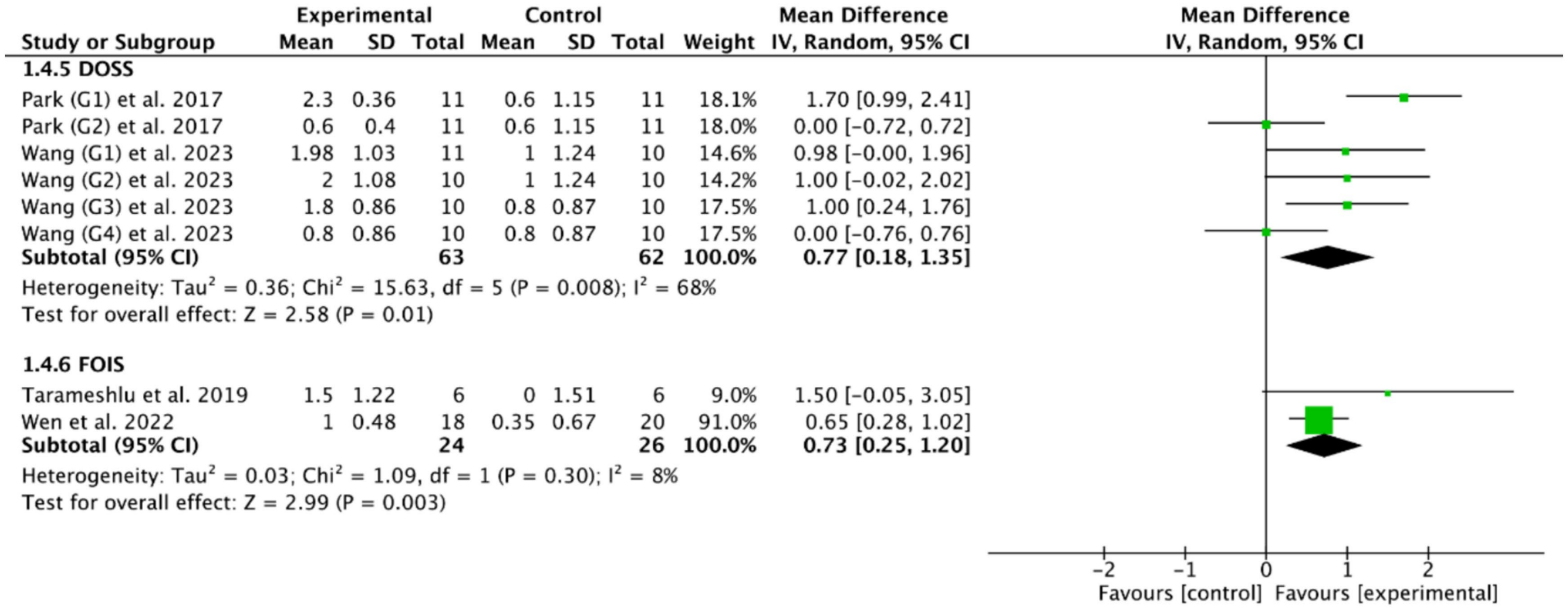
Efficacy of cerebral rTMS evaluated by the DOSS and FOIS scales.

Fourteen RCTs (27, 28, 33, 36–40, 47, 48, 50, 53–55) evaluated the efficacy of cerebral rTMS with mylohyoid motor cortex stimulation by seven standardized metrics: the PAS in nine RCTs (28, 37–40, 52–55), the SSA in five RCTs (27, 36, 50, 53, 55), the DD in three RCTs (27, 47, 48), the VDS in two RCTs (28, 39), the DOSS in two RCTs (28, 55), the FOIS in two RCTs (33, 52), and the WST in only one RCT (27). Figures 4, 5 demonstrated that cerebral rTMS improved the swallowing function evaluated by the WST (MD = −1.48, 95% CI [−2.46, −0.50], *p* < 0.05), the PAS (MD = −1.65, 95% CI [−2.42, −0.88], *p* < 0.05), the SSA (MD = −1.63, 95% CI [−2.62, −0.65], *p* < 0.05), the DD (MD = −1.37, 95% CI [−1.79, −0.95], *p* < 0.05), the FOIS (MD = 0.73, 95% CI [0.25, 1.20], *p* < 0.05), and the DOSS (MD = 0.77, 95% CI [0.18, 1.35], *p* < 0.05) scales, but did not improve the VDS scale (MD = −9.19, 95% CI [−21.03, 2.65], *p* = 0.13) compared with control group.

#### 3.4.1 Stimulation frequency

**Figure 6:**
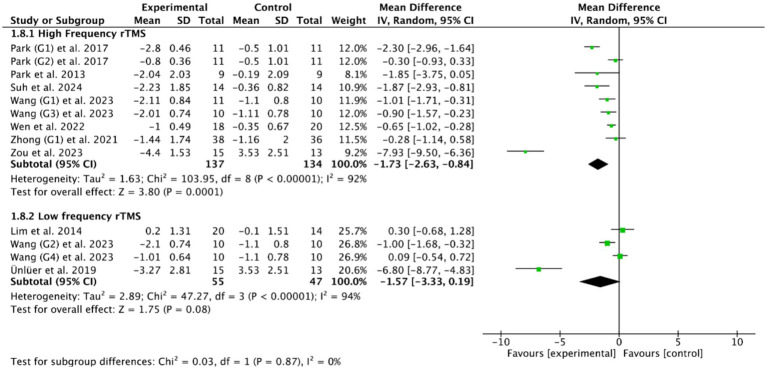
Efficacy of HF-rTMS versus LF-rTMS.

Among the 14 RCTs of cerebral rTMS treatment, HF-rTMS (3 Hz, 5 Hz, 10 Hz, 50 Hz) was applied in 9 RCTs (28, 36, 39, 40, 47, 48, 50, 53, 54), LF-rTMS (1 Hz) was applied in 3 RCTs (33, 37, 38), and another 2 RCTs (27, 55) included both HF-rTMS and LF-rTMS. Based on the common outcome indicator PAS (28, 37–40, 52–55), subgroup analysis showed that HF-rTMS (MD = −1.73, 95% CI [−2.63, −0.84], *p* < 0.05, Figure 6) could effectively improve the swallowing function of PSD patients compared with controls, while LF-rTMS (MD = −1.57, 95% CI [−3.33, 0.19], *p* > 0.05, Figure 6) failed to improve the swallowing function of PSD patients. However, there was no statistically significant difference between HF-rTMS and LF-rTMS (*p* = 0.87) in improving the swallowing function of patients with PSD.

#### 3.4.2 Stimulation site

**Figure 7:**
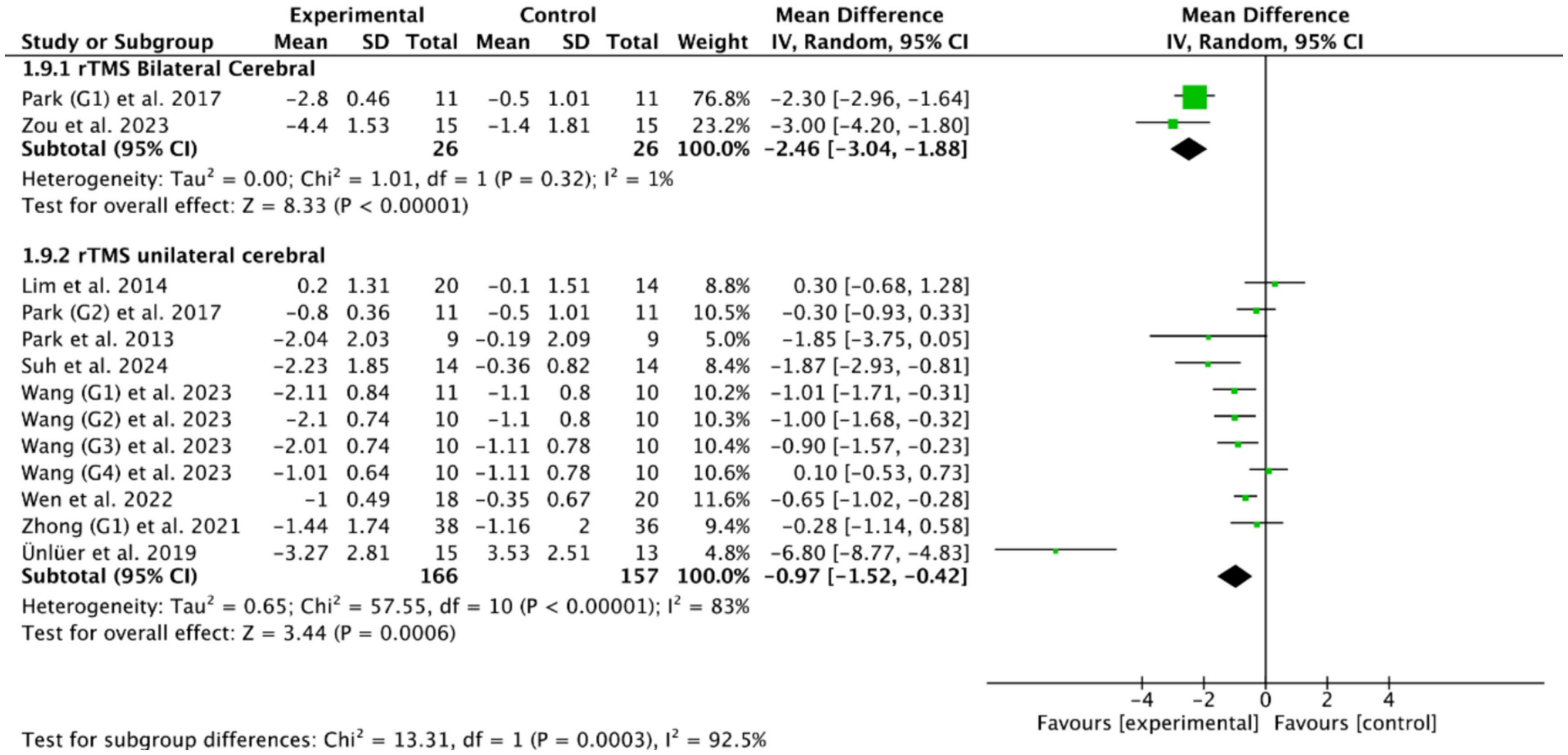
Efficacy of bilateral versus unilateral cerebral rTMS.

The stimulation site is also an important parameter worthy of attention in the rTMS treatment for patients with PSD. Among the 14 included RCTs, unilateral cerebral rTMS was used in 11 RCTs (27, 33, 36–39, 48, 50, 53–55), and bilateral cerebral rTMS was used in 3 RCTs (28, 40, 47). Based on the common outcome indicator PAS, meta-analysis showed that both bilateral cerebral rTMS (MD = −2.46, 95% CI [−3.04, −1.88], *p* < 0.05, Figure 7) and unilateral cerebral rTMS (MD = −0.97, 95% CI [−1.52, −0.42], *p* < 0.05, Figure 7) could effectively improve the swallowing function of patients with PSD compared with the control group. Furthermore, bilateral cerebral rTMS was more effective than unilateral cerebral rTMS in improving the swallowing function of patients with PSD (*p* < 0.05).

#### 3.4.3 Stimulation intensity

The stimulation intensity is also an important parameter of rTMS. Currently, researchers usually set the stimulation intensity as a percentage of the Resting Motor Threshold (RMT). Fourteen RCTs of cerebral rTMS treatment selected different stimulation intensities, such as 80% RMT, 90% RMT, 100% RMT, 110% RMT, 120% RMT, and 130% RMT. Among them, the stimulation intensities of 80% RMT, 110% RMT, and 130% RMT were each adopted by only one RCT (47, 50, 53). The stimulation intensities of 90% RMT was adopted by five RCTs (27, 28, 38–40), the stimulation intensities of 100% RMT was adopted by three RCTs (27, 37, 55), and the stimulation intensities of 120% RMT was adopted by three RCTs (33, 36, 48). Additionally, the researcher of one RCT adopted the active motor threshold (AMT) and selected 80% AMT as the stimulation intensity (54). These studies all reported that cerebral rTMS with different stimulation intensities could effectively improve the swallowing function in patients with PSD.

### 3.5 Efficacy of cerebellar rTMS

**Figure 8:**
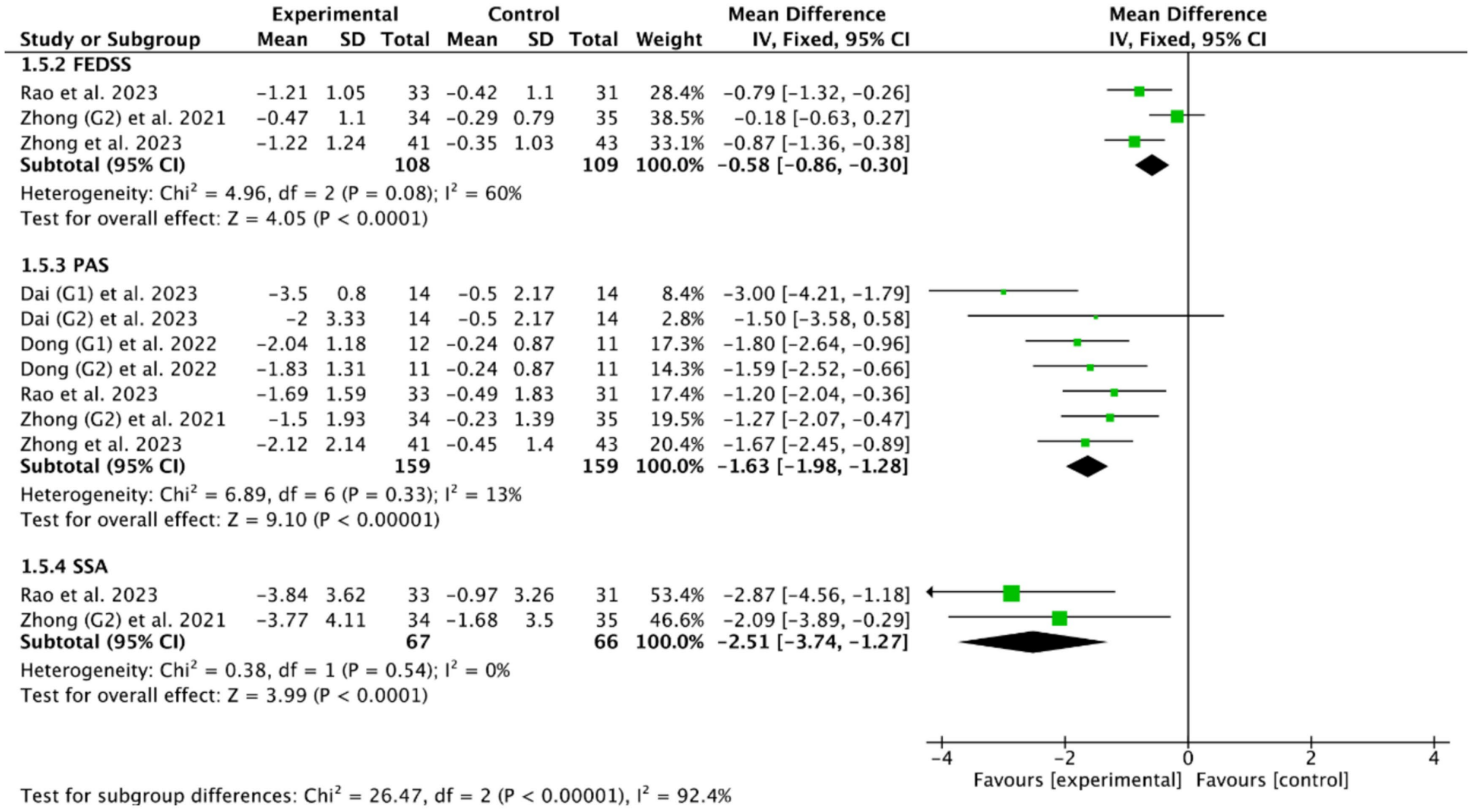
Efficacy of cerebellar rTMS assessed by the FEDSS, PAS, and SSA scales.

**Figure 9:**
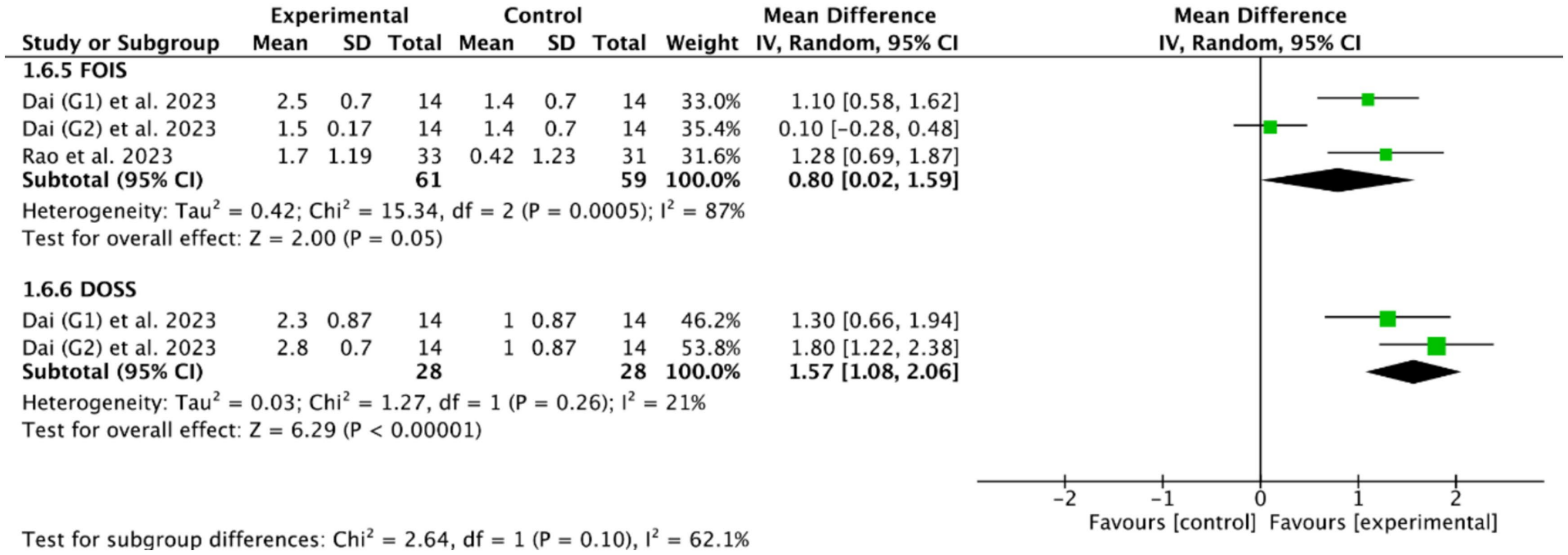
Efficacy of cerebellar rTMS assessed by the FOIS and DOSS sacles.

Among the 18 RCTs we included, 5 RCTs (46, 49, 51, 53, 56) evaluated the efficacy of cerebellar rTMS in patients with PSD by five standardized dysphagia metrics, such as the FEDSS in 3 RCTs (49, 51, 53), the PAS in 5 RCTs (46, 49, 51, 53, 56), the SSA in 2 RCTs (49, 53), the FOIS in 2 RCTs (46, 49), and the DOSS in only one RCT (46). Figures 8, 9 showed that cerebellar rTMS treatment could effectively improve the swallowing function of patients with PSD (FEDSS: MD = −0.58, 95% CI [−0.86, −0.30], *p* < 0.05; PAS: MD = −1.63, 95% CI [−1.98, −1.28], *p* < 0.05; SSA: MD = −2.51, 95% CI [−3.74, −1.27], *p* < 0.05; FOIS: MD = 0.80, 95% CI [0.02, 1.59], *p* = 0.05; DOSS: MD = 1.57, 95% CI [1.08, 2.06], *p* < 0.05).

#### 3.5.1 Stimulation frequency

**Figure 10:**
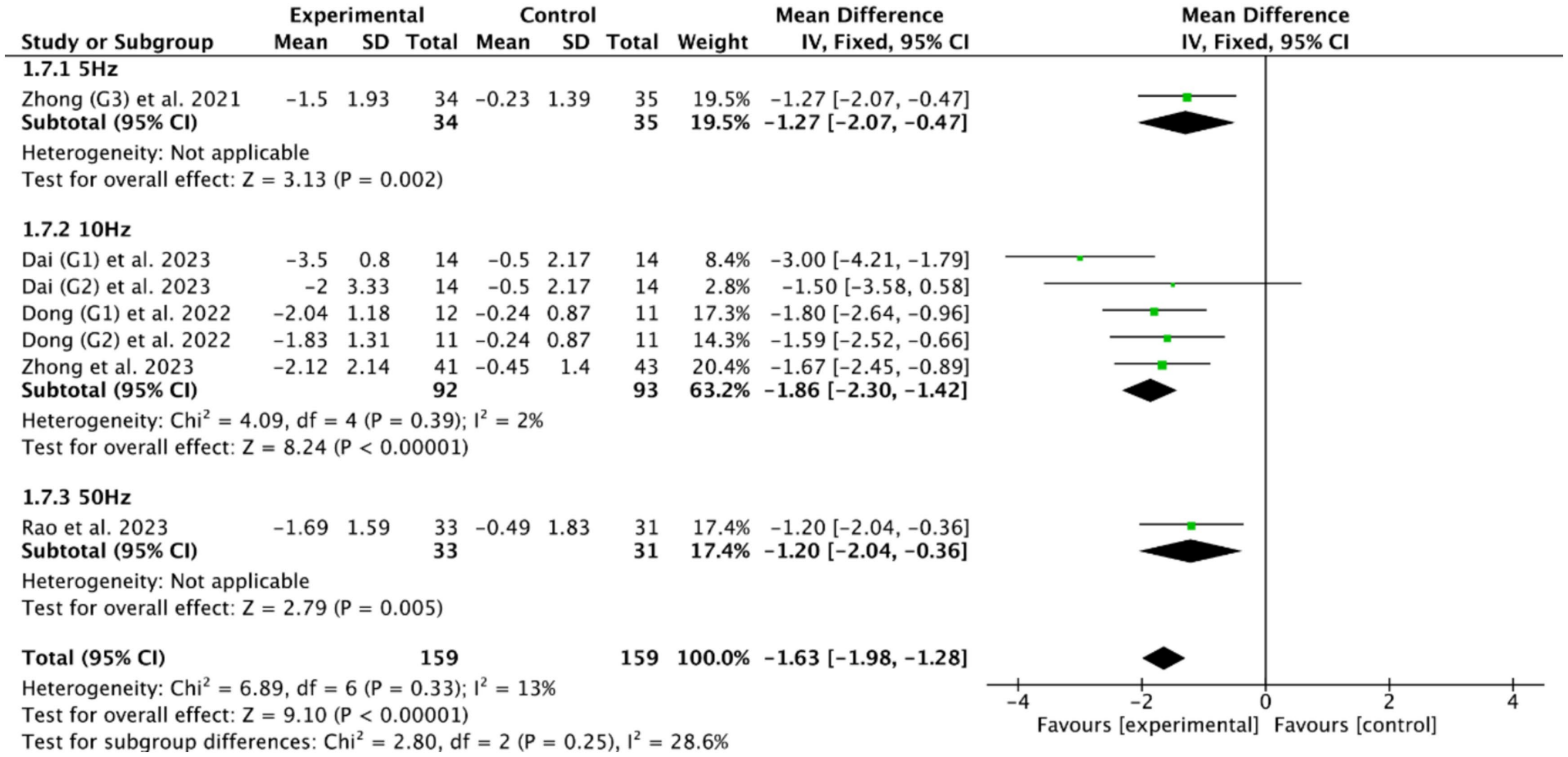
Efficacy of cerebellar rTMS with different stimulation frequency evaluated by PAS score.

Among the five RCTs (46, 49, 51, 53, 56) of cerebellar rTMS treatment, three RCTs (46, 51, 56) utilized a stimulation frequency of 10 Hz, one RCT (53) employed 5 Hz, and one RCT (49) implemented a specialized protocol using intermittent theta burst stimulation (iTBS) at 50 Hz. Based on the commonly used outcome indicator, the PAS, the meta-analysis showed that cerebellar rTMS at 5 Hz (MD = −1.27, 95% CI [−2.07, −0.47], *p* < 0.05), cerebellar rTMS at 10 Hz (MD = −1.86, 95% CI [−2.30, −1.42], *p* < 0.05), and cerebellar rTMS at 50 Hz (MD = −1.20, 95% CI [−2.40, −0.36], *p* = 0.05, Figure 10) could all effectively improve the swallowing function of patients with PSD compared with the control group.

#### 3.5.2 Stimulation site

**Figure 11:**
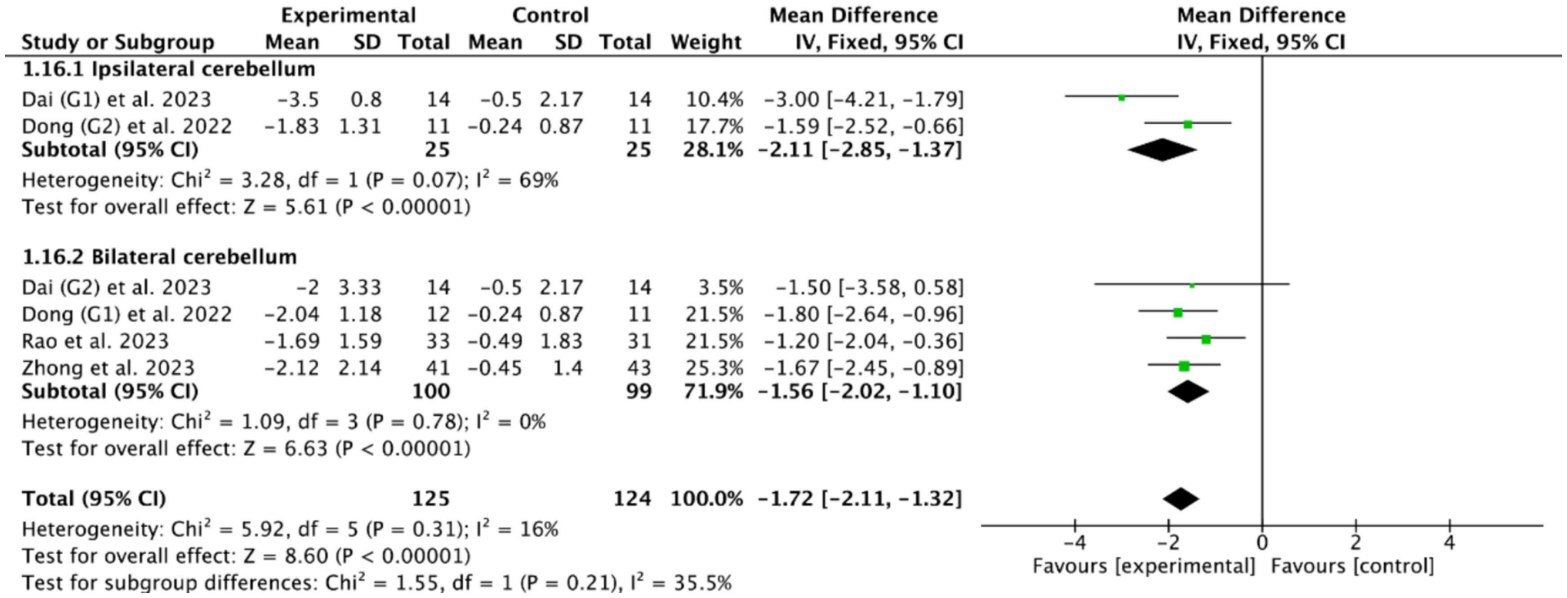
Efficacy of bilateral versus unilateral cerebellar rTMS evaluated by PAS score.

Concerning stimulation sites, four RCTs (46, 49, 51, 56) targeted the bilateral cerebellar swallowing functional area (with two of these concurrently investigating ipsilateral cerebellar stimulation), while one RCT (53) did not specify the stimulation location. Based on the commonly used outcome indicator, the PAS, the meta-analysis showed that ipsilateral cerebellar rTMS (MD = −2.11, 95% CI [−2.85, −1.37], *p* < 0.05) and bilateral cerebellar rTMS (MD = −1.56, 95% CI [−2.02, −1.10], *p* < 0.05, Figure 11) could both effectively improve the swallowing function of patients with PSD compared with the control group.

#### 3.5.3 Stimulation intensity

Regarding stimulation intensity, the included studies demonstrated variability in intensity: two trials applied 80% RMT (51, 56), with the remaining three employing 90% RMT (46), 100% RMT (49), and 110% RMT (53) respectively. These studies all reported that cerebellar rTMS with different stimulation intensities could effectively improve the swallowing function in patients with PSD.

### 3.6 The comparison of therapeutic effects between cerebellar and cerebral rTMS

**Figure 12:**
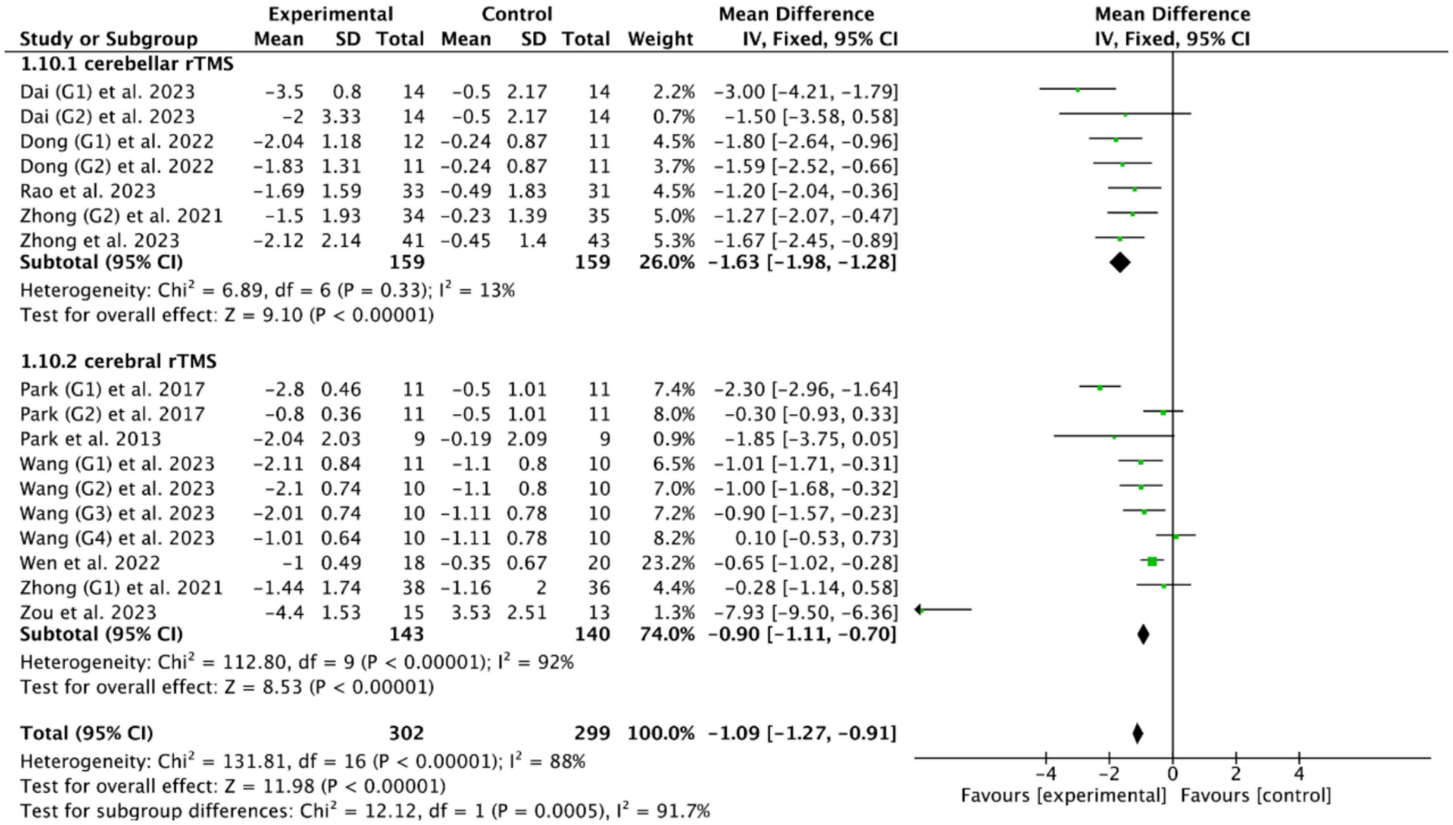
Efficacy of cerebellar versus cerebral rTMS evaluated by the PAS score.

Among the included studies, PAS and DOSS were the most common outcome indicators for PSD: 6 RCTs (28, 39, 40, 52, 53, 55) of cerebral rTMS used PAS as the outcome indicator, and 5 RCTs (46, 49, 51, 53, 56) of cerebellar rTMS used PAS as the outcome indicator; 2 RCTs (28, 55) of cerebral rTMS used DOSS as the outcome indicator, and one RCT (46) of cerebellar rTMS used DOSS as the outcome indicator. The subgroup analysis of 10 RCTs (28, 39, 40, 46, 49, 51–53, 55, 56) based on the PAS outcome indicator showed that both cerebellar rTMS (MD = −1.63, 95% CI [−1.98, −1.28], *p* < 0.05) and cerebral rTMS (MD = −0.90, 95% CI [−1.11, −0.70], *p* < 0.05, Figure 12) could effectively improve the swallowing function of patients compared with the control group. Furthermore, cerebellar rTMS was more effective in promoting the recovery of swallowing function than cerebral rTMS (*p* < 0.05).

**Figure 13:**
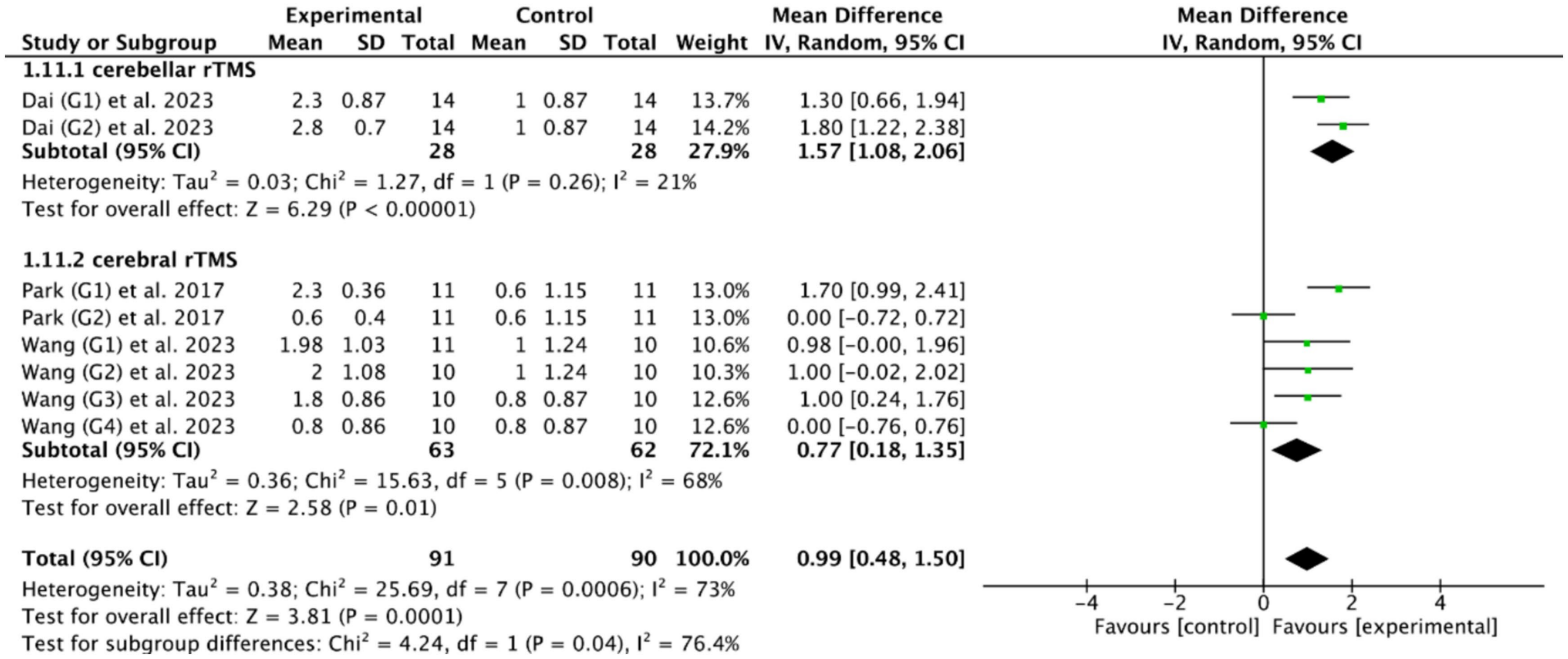
Efficacy of cerebellar versus cerebral rTMS evaluated by DOSS score.

Based on the DOSS outcome indicator, the subgroup analysis of 3 RCTs (28, 46, 55) showed that both cerebellar rTMS (MD = 1.57, 95% CI [1.08, 2.06], *p* < 0.05) and cerebral rTMS (MD = 0.77, 95% CI [0.18, 1.35], *p* < 0.05, Figure 13) could effectively improve the swallowing function of patients with PSD compared with the control group. Moreover, cerebellar rTMS was superior to cerebral rTMS improve the swallowing function of patients with PSD (*p* = 0.04).

### 3.7 Adverse event

Among the 18 included RCTs, only 5 RCTs (37, 38, 53, 55, 56) reported that a small number of patients (< 5%) experienced transient adverse reactions, such as headache, hearing loss, dizziness, and nosebleeds during the trial. And no subject withdrew from the trial study due to severe adverse reactions.

## 4 Discussion

### 4.1 The mechanism of rTMS in the treatment of PSD

Cerebral motor cortex and cerebellum are the commonly used stimulation sites of rTMS treatment for PSD. In this study, we included 18 RCTs and found that both cerebral and cerebellar rTMS treatment can effectively and safely improve the swallowing function of PSD patients. This is consistent with previous systematic reviews and meta-analyses (36, 57). For example, Wen et al. (36) included 463 PSD patients from 11 RCTs and revealed that cerebral rTMS treatment could effectively improve the swallowing function of patients with PSD. Similarly, after analyzing 5 RCTs of cerebellar rTMS for the treatment of PSD, Liu et al. (57) also concluded that cerebellar rTMS treatment could effectively improve the swallowing function of patients. Unlike previous findings, our study demonstrated that cerebellar rTMS yielded superior therapeutic effects compared to cerebral rTMS, which may be attributed to differences in their underlying mechanisms.

The normal functioning of swallowing relies on the coordinated action of multiple muscle groups in the face, oropharynx, and esophagus, with its neural control involving a complex network system comprising the cerebral cortex, brainstem, and cerebellum. At the cerebral cortical level, its primary function lies in initiating and regulating voluntary swallowing processes while finely controlling the oropharyngeal phase (58). When stroke damages the cortical swallowing centers, the brain’s ability to control swallowing muscles weakens, leading to swallowing dysfunction. rTMS exerts therapeutic effects by directly modulating cortical excitability: HF-rTMS enhances neuronal excitability in the cortex, inducing long-term potentiation (LTP) effects and activating dormant neural pathways to promote motor signal transmission. In contrast, LF-rTMS suppresses overactive regions, eliciting long-term depression (LTD) effects and restoring balance in brain activity (22). Additionally, rTMS can regulate the metabolism of neurotransmitters such as gamma-aminobutyric acid (GABA), glutamate, and dopamine (59), improving neural function by altering the excitatory-inhibitory balance. Notably, rTMS also promotes the secretion of brain-derived neurotrophic factor (BDNF) (60), which plays a critical role in synaptic plasticity and neuronal survival, facilitating the reconstruction of damaged neural networks.

The cerebellum plays a unique role in swallowing control. Unlike the direct regulation by the cerebral cortex, the cerebellum participates in the fine-tuning of swallowing through modular neural circuits. Although motor commands originate in the cerebral cortex, the cerebellum is crucial for ensuring the accuracy, coordination, and fluidity of muscle activity (61, 62). Anatomically, the cortico-cerebellar tracts transmit neural signals from the motor and sensory cortices to the cerebellum, enabling it to influence multiple swallowing-related neural circuits, including the primary motor area, supplementary motor area, sensory cortex, and cingulate gyrus (63). This parallel operation of multiple circuits suggests that the neural control of swallowing is achieved through interconnected modular networks. Particularly noteworthy is that cerebellar rTMS can not only modulate swallowing function through these neural pathways but also directly stimulate muscle groups involved in swallowing (64, 65), offering a novel therapeutic target for PSD.

### 4.2 Efficacy of cerebral rTMS with different stimulation parameters

Firstly, stimulation frequency is an important stimulation parameter of rTMS. In the studies we included, different stimulation frequencies were used for cerebral rTMS treatment, including 1 Hz (27, 33, 37, 38, 55), 3 Hz (27, 47, 48), 5 Hz (28, 39, 40, 52, 55), and 50 Hz (50, 54). These studies all reported that cerebral rTMS with different stimulation frequencies could effectively improve the swallowing function in patients with PSD. Furthermore, Meta-analyses showed that HF-rTMS could improve the swallowing function of PSD patients, while LF-rTMS failed to improve the swallowing function of PSD patients compared with controls. Notably, there is no significant difference between HF-rTMS and LF-rTMS in the treatment of PSD, which might be attributable to limited numbers of studies.

Secondly, stimulation site also plays a key role in the treatment of rTMS. In this study, we found that both bilateral cerebral rTMS and unilateral cerebral rTMS could effectively improve the swallowing function of patients with PSD. Notably, bilateral cerebral rTMS was more effective than unilateral cerebral rTMS in the treatment of PSD, probably because swallowing function is controlled by cerebral motor cortex bilaterally (66, 67).

Thirdly, stimulation intensity is another important intervention parameter of rTMS. Different stimulation intensities such as 80% RMT (50), 90% RMT (27, 28, 38–40), 100% RMT (37, 55), 120% RMT (33, 48, 52), and 130% RMT (47), were used for rTMS treatment. These studies all reported that cerebral rTMS with different stimulation intensities could effectively improve the swallowing function in patients with PSD. Current international guidelines of rTMS recommend controlling stimulation intensity in the range of 80–120% of RMT to optimize efficacy while minimizing adverse effects (18).

### 4.3 Efficacy of cerebellar rTMS with different stimulation parameters

In the studies we included, different stimulation frequencies of cerebellar rTMS were also used for the treatment of PSD, including 5 Hz (53), 10 Hz (46, 51, 56), and 50 Hz (49). All these studies reported that cerebellar rTMS with different stimulation frequencies could effectively improve the swallowing function of PSD patients. A study tested the effects of bilateral cerebellar rTMS with different stimulation frequencies (5 Hz, 10 Hz, and 20 Hz) on swallowing function, and the results showed that only the stimulation frequency of 10 Hz could significantly enhance the amplitude of pharyngeal evoked potentials in the bilateral cerebellar swallowing network (65). Our analysis also showed that 10 Hz is currently the main stimulation frequency of cerebellar rTMS.

In addition, studies have shown that rTMS stimulating either side of the cerebellum can improve the swallowing function of patients (68, 69). Four of five included RCTs (46, 49, 51, 56) adopted bilateral cerebellar targets with consistent positive outcomes. Two of these studies (46, 56) compared the efficacy differences between unilateral and bilateral cerebellar rTMS, and the results revealed that bilateral stimulation was superior to unilateral stimulation, which might be mediated through two mechanisms: (1) Bilateral stimulation enhances functional connectivity across cerebello-cortical pathways, synchronously amplifying cortical excitability in bilateral swallowing networks; (2) Simultaneous activation of bilateral orolingual muscle representations compensates for cortical disconnection, facilitating neuromuscular re-education. These neurophysiological advantages make the bilateral cerebellum an optimal stimulation target of rTMS treatment for PSD.

In the studies we included, different stimulation intensities of cerebellar rTMS were used, including 80% RMT (51, 56), 90%RMT (46), 100%RMT (49), and 110%RMT (53). Notable heterogeneity exists in stimulation intensities across studies, precluding definitive subgroup comparisons. Neuroanatomically, the cerebellum’s extensive connectivity with motor systems raises concerns about potential exacerbation of spasticity through excessive stimulation intensity. Interestingly, our analysis revealed comparable efficacy at 80% RMT, suggesting lower-intensity protocols may balance therapeutic effect and tolerability. However, the optimal intensity requires further investigation through dedicated cerebellar rTMS trials.

### 4.4 Efficacy of cerebellar rTMS versus cerebral rTMS

To our best knowledge, there are no studies to directly compare the efficacy of cerebral and cerebellar rTMS in the treatment of PSD. In this study, subgroup analysis revealed that cerebellar rTMS appears to be superior to cerebral rTMS in the treatment of PSD evaluated by both PAS and DOSS scores. This differential effect may be attributed to the cerebellum’s unique position in motor network hierarchy—its stimulation simultaneously modulates corticospinal excitability via thalamic relays while directly activating pharyngeal muscle representations (64, 65). Furthermore, cerebellar neuroplasticity mechanisms appear less compromised by stroke-related white matter damage, providing alternative pathways for functional recovery (70). Notably, these promising findings require cautious interpretation due to limited RCTs, which should be verified in the future RCTs.

## 5 Limitations

This study has several limitations. Firstly, the sample size of the included RCTs was small. A too small sample size can easily lead to biases when assessing the therapeutic effect of rTMS on PSD. Secondly, all the included RCTs involved different types of stroke patients, and the lesion sites of stroke were not consistent, but we were unable to conduct further subgroup analyses. Lastly, the RCTs were limited to those published in English, and no more in-depth search was conducted for RCTs in other languages, so there may be publication bias to a certain extent.

## 6 Conclusion

This study showed that both cerebral and cerebellar rTMS treatment can effectively and safely improve the swallowing function of PSD patients. Furthermore, cerebellar rTMS appears to be superior to cerebral rTMS in the treatment of PSD, which should be verified in the future RCTs.

## Data Availability

The original contributions presented in the study are included in the article/supplementary material, further inquiries can be directed to the corresponding authors.
